# LarvaTagger: manual and automatic tagging of *Drosophila* larval behaviour

**DOI:** 10.1093/bioinformatics/btae441

**Published:** 2024-07-05

**Authors:** François Laurent, Alexandre Blanc, Lilly May, Lautaro Gándara, Benjamin T Cocanougher, Benjamin M W Jones, Peter Hague, Chloé Barré, Christian L Vestergaard, Justin Crocker, Marta Zlatic, Tihana Jovanic, Jean-Baptiste Masson

**Affiliations:** Institut Pasteur, Université Paris Cité, CNRS UMR 3571, Decision and Bayesian Computation, 75015 Paris, France; Épiméthée, INRIA, 75015 Paris, France; Institut Pasteur, Université Paris Cité, Bioinformatics and Biostatistics Hub, F-75015 Paris, France; Institut Pasteur, Université Paris Cité, CNRS UMR 3571, Decision and Bayesian Computation, 75015 Paris, France; Épiméthée, INRIA, 75015 Paris, France; Institut Pasteur, Université Paris Cité, CNRS UMR 3571, Decision and Bayesian Computation, 75015 Paris, France; TUM School of Computation, Information and Technology, 80333 Munich, Germany; European Molecular Biology Laboratory, Developmental Biology, 69117 Heidelberg, Germany; Department of Zoology, University of Cambridge, Cambridge CB2 3EJ, United Kingdom; Janelia Research Campus, Howard Hughes Medical Institute, Ashburn, VA 20147, United States; MRC Laboratory of Molecular Biology, Cambridge CB2 0QH, United Kingdom; Department of Zoology, University of Cambridge, Cambridge CB2 3EJ, United Kingdom; Janelia Research Campus, Howard Hughes Medical Institute, Ashburn, VA 20147, United States; MRC Laboratory of Molecular Biology, Cambridge CB2 0QH, United Kingdom; Department of Zoology, University of Cambridge, Cambridge CB2 3EJ, United Kingdom; Janelia Research Campus, Howard Hughes Medical Institute, Ashburn, VA 20147, United States; MRC Laboratory of Molecular Biology, Cambridge CB2 0QH, United Kingdom; Institut Pasteur, Université Paris Cité, CNRS UMR 3571, Decision and Bayesian Computation, 75015 Paris, France; Épiméthée, INRIA, 75015 Paris, France; Institut Pasteur, Université Paris Cité, CNRS UMR 3571, Decision and Bayesian Computation, 75015 Paris, France; Épiméthée, INRIA, 75015 Paris, France; European Molecular Biology Laboratory, Developmental Biology, 69117 Heidelberg, Germany; Department of Zoology, University of Cambridge, Cambridge CB2 3EJ, United Kingdom; Janelia Research Campus, Howard Hughes Medical Institute, Ashburn, VA 20147, United States; MRC Laboratory of Molecular Biology, Cambridge CB2 0QH, United Kingdom; Institut des Neurosciences Paris-Saclay, Université Paris-Saclay, Centre National de la Recherche Scientifique, UMR 9197, 91400 Saclay, France; Institut Pasteur, Université Paris Cité, CNRS UMR 3571, Decision and Bayesian Computation, 75015 Paris, France; Épiméthée, INRIA, 75015 Paris, France

## Abstract

**Motivation:**

As more behavioural assays are carried out in large-scale experiments on *Drosophila* larvae, the definitions of the archetypal actions of a larva are regularly refined. In addition, video recording and tracking technologies constantly evolve. Consequently, automatic tagging tools for *Drosophila* larval behaviour must be retrained to learn new representations from new data. However, existing tools cannot transfer knowledge from large amounts of previously accumulated data. We introduce LarvaTagger, a piece of software that combines a pre-trained deep neural network, providing a continuous latent representation of larva actions for stereotypical behaviour identification, with a graphical user interface to manually tag the behaviour and train new automatic taggers with the updated ground truth.

**Results:**

We reproduced results from an automatic tagger with high accuracy, and we demonstrated that pre-training on large databases accelerates the training of a new tagger, achieving similar prediction accuracy using less data.

**Availability and implementation:**

All the code is free and open source. Docker images are also available. See gitlab.pasteur.fr/nyx/LarvaTagger.jl.

## 1 Introduction

*Drosophila* larva is increasingly used as an animal model for large-scale experiments in behavioural neuroscience (Ohyama *et al.* 2013, Vogelstein *et al.* 2014, Jovanic *et al.* 2016, Masson *et al.* 2020). Such assays require automating multiple processing steps, from preprocessing the video streams to generating a behavioural readout suitable for comparing experimental conditions and interpreting the observed differences.

Characterizing the behaviour of highly deformable blob-shaped animals such as larvae has been challenging, in contrast to animals that exhibit distinguishable limbs, extensions or body parts. For example, deep learning techniques have been notably successful in identifying behavioural patterns, sometimes even without expert annotation, by tracking the distinct parts of structured-bodied animals (Mathis and Mathis 2020).

In large-scale behavioural experiments, a camera grabs the arena from above and shows a dorsal view of several dozen of *Drosophila* larvae. In most cases, their body segments are not clearly distinguishable. The limited information on the shape of individual larvae and the large amount of video data has motivated a multi-stage approach to behaviour extraction. Tracking procedures are applied first, often in the online regime, so only dynamic contours and spines are saved without the original video stream. Behaviour characterization is a separate offline processing step, where a time series of body postures is used as input.

Behaviour is typically characterized by such time series with an implicit function in mind: moving forward, turning, withdrawing, etc. Movement amplitude and speed are useful features to distinguish between actions. However, suppose the presumed function of the behaviour is given priority over the continuous representation of the motion. In that case, an expert may identify the same behaviour at different space and time scales.

Different experimental protocols will rely on using *Drosophila melanogaster* strains with different genetic backgrounds and/or various genetically modified lines that can exhibit different behavioral characteristics with similar behavioral categories displaying various dynamics, amplitudes, duration, and sometimes sequences. In addition, environmental context, like the crawling surface or temperature, will also influence larval behavior. It is thus necessary to be able to classify behavioural actions considering these different types of behavioural variation.

For example, a hunch, a type of defensive action that larvae perform typically in response to mechanical stimuli by retracting their head, is characterized by a faster head retraction than the otherwise similar movement of the head and thoracic segments during a peristaltic crawl. Time-dependent features would naturally be selected to distinguish a hunch from a specific phase of the peristaltic wave. However, some genetically modified larvae are slower in both types of movements, so their hunches are slower than other larvae’s peristaltic waves. Consequently, in this latter case, the successful identification of a hunch would preferably rely on duration-invariant features.

Generally speaking, each experimental paradigm introduces different constraints on characterizing behaviour. This makes the case for a deep-learning approach to action identification, with discriminating features automatically extracted from the raw time series of postures on a per-experiment basis.

We introduce LarvaTagger, a software tool for action identification based on a pre-trained neural network that can be retrained on new data and actions. LarvaTagger also features a graphical user interface to visualize tracking data and manually tag actions. The first section of this paper briefly lists key features and use cases for LarvaTagger. We discuss its contribution to the software ecosystem and the technologies it is based on. In the second section, we characterize the performance of a tagger trained to reproduce the behavioural readout of a widely used tagger (Masson *et al.* 2020), with similar changes in action probabilities for most larva lines. We also demonstrate transfer learning by first pre-training a neural network on a database in a self-supervised fashion and, second, training for a particular tagging task on another database.

Throughout the present article, we also make a general argument in favour of continuous representations of the observed behaviour as a complementary approach to the discrete behavioural readout given in terms of actions. In particular, representation spaces spanned by automatically extracted features—instead of predefined features such as speeds, angles, etc.—can be useful to design statistical tests (Blanc *et al.* 2024). In *Drosophila* larvae, several studies used similar representation spaces as state spaces (Carreira-Rosario *et al.* 2021, York *et al.* 2021), but no software is available yet to help implement these approaches, apart from MaggotUBA (Blanc *et al.* 2024). The conclusion stresses how LarvaTagger can facilitate the combination of both approaches.

## 2 Software elements and methods

LarvaTagger is a behaviour tagging tool for *Drosophila* larvae. It performs both manual and automatic tagging, and new taggers can be trained using a pre-trained neural network and labelled/tagged ‘ground-truth’ data.

The LarvaTagger project is divided into multiple sub-projects to support different use cases. For example, the user interface (UI) is provided by LarvaTagger.jl, available at gitlab.pasteur.fr/nyx/larvatagger.jl. The LarvaTagger.jl repository is the main entry point of the whole project and its documentation. However, the automatic tagging logic is functionally independent from the UI. We expect this to make the design of alternative taggers easier. In the present article, LarvaTagger is demonstrated in combination with a tagger based on an autoencoder known as MaggotUBA (Blanc *et al.* 2024).

LarvaTagger is preferably downloaded and run as a Docker image from Docker Hub (hub.docker.com/repository/docker/flaur/larvatagger). Past versions are available to ensure a high degree of reproducibility. Standalone scripts for Windows (cmd and PowerShell), macOS and Linux help operate the Docker image.

**Figure 1.**
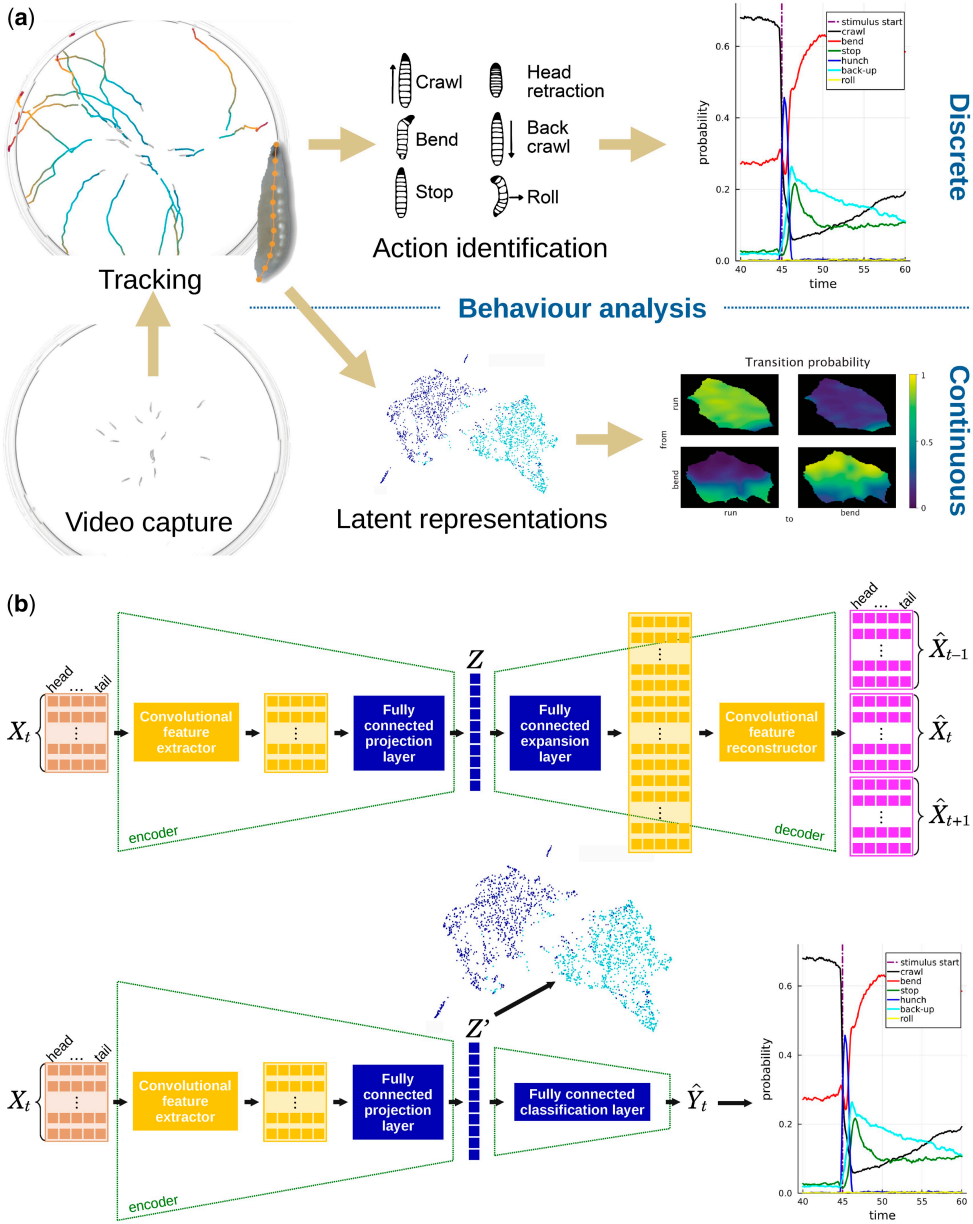
(a) Behaviour analysis pipelines. Behaving larvae are video-grabbed and tracked, which results in trajectory data together with shape data at each time point of a trajectory. The analysis of such data typically relies on identifying actions from a dictionary of actions, again at each time step of a larva’s trajectory. The resulting discrete behavioural readout can be compared between populations of larvae in terms of action proportions or sequences (top-right plot of action probability versus time). Alternatively, tracking data can be projected into a common feature space, with track segments represented as points. The feature space, also referred to as latent space (bottom-centre illustration of time segments as points in a 2D latent space), is first generated in an unsupervised fashion using large amounts of unlabelled data. Such representations can be used to design statistical tests or any analysis that involves groups or categories. As an example, transition probabilities between two actions are illustrated in a 2D latent space. LarvaTagger implements both action identification and generation of latent representations using a MaggotUBA-based tagger. (b) Integration of MaggotUBA (top diagram), an autoencoder that extracts features of the continuous behaviour in a self-supervised fashion. A sequence of postures *X_t_* (or track segment) is compressed into a low-dimensional latent representation *Z* from which a longer sequence, including past and future postures in addition to the input *X_t_*, is reconstructed. Training the autoencoder allows learning behavioural features that compress the dynamics in the latent space. The encoder features are reused in combination with a classification stage in the MaggotUBA-based tagger (bottom diagram) to learn a discrete behavioural dictionary. The MaggotUBA-based tagger that LarvaTagger embarks can be used to generate both types of behavioural readouts.

Figure 1a identifies other pieces of software LarvaTagger can be combined with or that it can replace. In particular, the original motivation for LarvaTagger was to replace Pipeline_action_analysis_t5_pasteur_janelia (referred to in the following as Pipeline_pasteur_janelia, Masson *et al.* 2020), a tagger that identifies specific actions but cannot be adjusted to new data or new actions.

### 2.1 Tracking

LarvaTagger supports several data formats of tracking data, including spine/outline files from MWT/Choreography (Swierczek *et al.* 2011), table.csv files from FIMTrack v2 (Risse *et al.* 2017) and MatLab files from Pipeline_pasteur_janelia (Masson *et al.* 2020). File converters have also been written for HDF5 files from Tierpsy Tracker (Javer *et al.* 2018; see also github.com/Tierpsy/tierpsy-tracker) and DeepLabCut (Lauer *et al.* 2022).

Tracking tools must be suitable for *Drosophila* larvae. They should generate point tracks and posture data, particularly the larval mid-line for automatic tagging purposes and the contour for visualization and manual tagging purposes. MAGAT is another example of tracking software that, like most tracking suites, comes with a package (MAGAT Analyzer) for extracting common features such as directions, speeds, angles, etc. (Gershow *et al.* 2012). While such predefined features could be (and were) used to identify actions, they are not used by LarvaTagger.

Tracking *Drosophila* larvae is challenging because of the lack of structure in the 2D shape of a larva when seen from above. Furthermore, the choice of the number of larvae per behavioural assay represents a trade-off between increasing statistics and minimizing crossing events. Indeed, the larvae have highly deformable bodies and are challenging to properly differentiate and track when they interact while being filmed at low resolutions [recent progress has been made at high resolution (Thane *et al.* 2023)]. All the tracking solutions we know of require precise calibration and may fail to detect moving objects with minor variations of experimental parameters.

While larva tracking is out of LarvaTagger’s scope, we leveraged mwt-container, an automatic tracking pipeline for the batch processing of video data. mwt-container is conveniently available as a Docker image, easily convertible into a Singularity/Apptainer image file for use on high-performance computing clusters. It takes AVI files as input and applies the Multi-Worm Tracker [specifically mwt-core and Choreography, Swierczek *et al.* (2011)]. Unlike other applications, MWT is shipped without its LabView components. Some tracking hyper-parameters are automatically adjusted so that the number of simultaneously tracked objects approaches the number of larvae in the assay. See Supplementary Section Tracking for more details.

### 2.2 Manual tagging

LarvaTagger’s entry point is the LarvaTagger.jl Julia package, available at gitlab.pasteur.fr/nyx/larvatagger.jl, which provides command-line (CLI) and graphical user interfaces (GUI). The GUI is suitable for inspecting and manually tagging tracking data. It features the following capabilities:

- Searching for a track/larva by id.
- Visualizing tracking data and any associated label on a per-frame basis or animated at different playback speeds. Labels are colour-coded, and the contour of the tracked larva is coloured accordingly.
- Assigning labels, for individual larvae at each defined time step or across entire time segments.
- Defining new labels, renaming labels, changing the associated colour, etc.
- Undoing all manual editions performed on a larva.
- Editing the metadata of the behavioural assay in a dedicated panel.
- Exporting the labelling/tagging information of selected tracks; the desired tracks can be selected from another panel indicating which tracks have been manually edited.

The labelling/tagging information is stored in a JSON file whose structure is very similar to the *Worm tracker Commons Object Notation* (WCON) format (Javer *et al.* 2018) for tracking data, with specifications available at gitlab.pasteur.fr/nyx/planarlarvae.jl/#json-files. JSON files are human-readable, suitable for storing metadata in addition to data, and easy to load and edit in every programming language or even using a text editor.

### 2.3 Automatic tagging

LarvaTagger can automatically tag the behaviour in discrete actions, either from the GUI for the currently loaded data file or in batch mode using the CLI. LarvaTagger separates the UI and general tagging API from the core logic of the tagger. This separation should make it easier to implement other tagging backends. By default in the Docker images, LarvaTagger ships with a tagging backend based on MaggotUBA, called MaggotUBA-adapter. In turn, this latter backend ships with a default tagger, which currently is called 20230311 and is demonstrated in section below.

The tagging backend supports a default tagger and can train new taggers with different target actions and ground-truth data. Conceptually, a tagger is an instance of the model implemented by the backend. Training a new tagger can only be performed using the CLI or the Python API of MaggotUBA-adapter.

The LarvaTagger project is divided into multiple sub-projects; some of these are listed below:

- LarvaTagger.jl: command-line and graphical user interfaces, which is the recommended entry point.
- TaggingBackends: backbone for operating and designing tagging backends; manages data preparation and trained taggers.
- MaggotUBA-adapter: implements a MaggotUBA-based tagger as illustrated in 1b; can be operated directly as a library in Python.
- PlanarLarvae.jl: low-level logic for handling data files and sampling in data repositories; specifies the JSON file format used to store tagging information and metadata.

A map of these sub-projects is found in the developer documentation available at gitlab.pasteur.fr/nyx/larvatagger.jl/-/blob/dev/doc/develop.md.

To our knowledge, the closest software tool around with manual tagging and retraining of automatic taggers is JAABA (Kabra *et al.* 2013). Other tools involved in studies that identified larval actions [e.g. MAGAT Analyzer in Gershow *et al.* (2012)] do not implement the actual action identification; they only extract features (or ‘metrics’) for which thresholds (or a decision strategy) still have to be defined. In addition, a key distinction between LarvaTagger and JAABA is MaggotUBA’s intermediate latent representations of the behaviour, which does not necessarily depend on data annotations or discrete actions as defined by behaviour experts. Indeed, as illustrated in Fig. 1b, LarvaTagger relies on MaggotUBA [Blanc *et al.* (2024), implemented with PyTorch (Paszke *et al.* 2019)], a self-supervised autoencoder that can project short sequences of postures into a common low-dimensional latent space. As showcased in Blanc *et al.* (2024), the latent representations can be used to implement a statistical test or inspected in regions of the latent space where higher between-group variance is observed.

LarvaTagger trains new taggers using these latent representations to feed a classifier with automatically extracted features. The latent representations can evolve if the training procedure is allowed to fine-tune the encoder neural network with the purpose of action classification tasks (Fig. 1b). In addition to identifying discrete actions, a MaggotUBA-based tagger can generate these latent representations for the input data. This can be performed using the CLI.

## 3 Emulating an existing tagger

As mentioned above, a motivation for LarvaTagger was to replace Pipeline_pasteur_janelia (Masson *et al.* 2020), a tagger that identifies specific actions. Indeed, this latter tagger cannot be retrained because it was incrementally designed following an active learning approach: predictive components were added to the tagger as new needs arose, e.g. new actions were to be identified, or corrections to be performed (de Tredern *et al.* 2023, Lehman *et al.* 2023). In addition, each component takes as input not only explicit features of the data, but also predictions from the preexisting components, thus forming a hierarchical ensemble of classifiers. While the hierarchical design has helped enforce priorities of some actions over others (in particular to handle some actions so rare as to be only identified in some controlled circumstances or genetic lines of *Drosophila*), adapting the tagger to new data or new actions is challenging, and may require substantial redesign.

Pipeline_pasteur_janelia identifies six actions (Masson *et al.* 2020): forward crawl (*crawl*), backward crawl (*back* or *back-up*), head cast or bend (*bend*), hunch, roll and stop. Each action can be further labelled as small or large (or weak or strong). The small/weak actions are grouped under a single *small action* class, resulting in a total of seven classes.

### 3.1 Reproducing qualitative results

In Masson *et al.* (2020), the authors found neurons of interest by inactivating them in an assay where they subjected larvae to an air puff as a mechanical stimulus (Jovanic *et al.* 2016). They identified these neurons by observing statistically significant differences in the frequency of actions and/or transitions between actions, in response to sensory stimulation. This comparison was made between larvae with inactivated neurons and reference larvae (*w;;attP2*). In total, 293 genetic lines were found to induce significant behavioural variations. These data come from a screening experiment in Jovanic *et al.* (2016) and Masson *et al.* (2020).

We trained a tagger (referred to as 20230311) based on MaggotUBA on data from the same optogenetic screen used to train the reference tagger in Masson *et al.* (2020). We analysed the same selected lines from Jovanic *et al.* (2016) and Masson *et al.* (2020) and mimicked the evaluation procedure of Masson *et al.* (2020), selecting a 1-s time window right after the stimulus onset at 45 s, and applying a similar statistical procedure ($\chi^{2}$ tests, Bonferroni-corrected for 471 comparisons), to characterize the increases or decreases in population probabilities for each action. The training and evaluation procedure is further detailed in Supplementary Sections The 20230311 tagger and Evaluation on Jovanic *et al.* (2016) and Masson *et al.* (2020).

**Figure 2.**
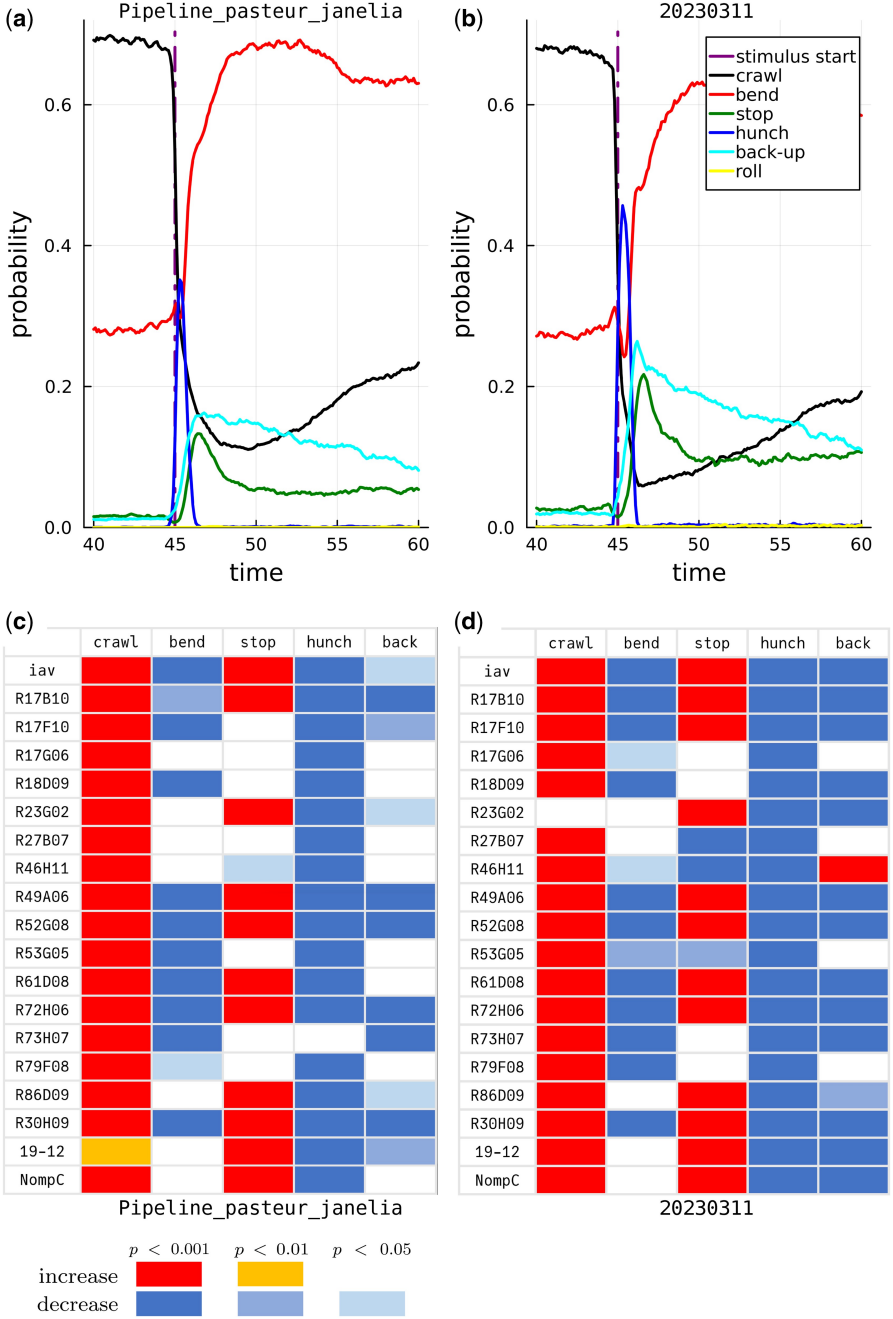
Left panels originate from Pipeline_pasteur_janelia. Right panels originate from 20230311. (a) and (b) Probability time series for the six actions of interest (*small action* not accounted for) in a population of control larvae (*w;;attP2*). At t=45s, the larvae received a 38-s long air puff, resulting in a dramatic change in behaviour reflected in the action probabilities over time. While baseline probabilities are well preserved in (b), as compared with (a), short-term response probabilities exhibit slightly more overall changes in (b), with more frequent hunches, back-ups and stops. (c) and (d) Significant differences in individual action probabilities between a selection of *Drosophila* lines and the control *w;;attP2* line. All *P*-values are Bonferroni-corrected for 471 comparisons. (c) is a reproduction of Fig. 3e in Masson *et al.* (2020). Some differences between (c) and (d) can be observed. In particular, a few effects in (c) are lost in (d), and, more frequently, effects observed with the 20230311 tagger (d) were not found using the original tagger (c).

Figure 2 illustrates side-by-side results from each tagger. For example, Fig. 2a and b show that the proportions of the different actions are well preserved in control larvae, especially before the stimulus onset. In response to the air puff, the overall change in proportions (or probabilities) is larger with the MaggotUBA-based tagger than with the original tagger. Figure 2c and d show how some *Drosophila* lines compare with the control line, in terms of increase or decrease in action probabilities, and how the MaggotUBA-based tagger reproduces most of these differences.

**Figure 3.**
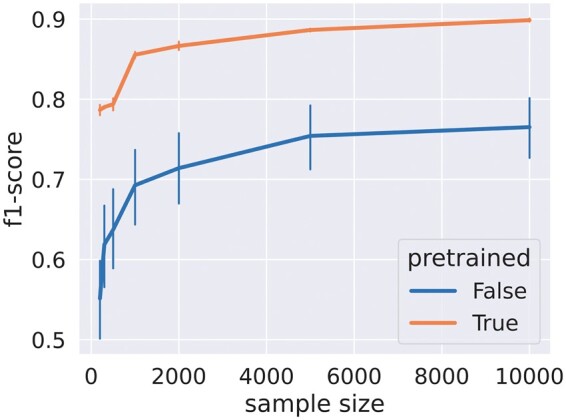
*f*1-scores for different training dataset sizes (abscissa), with the MaggotUBA encoder pre-trained (“True” curve) or not (“False” curve). Train and test datasets were drawn from the new optogenetic screen. Pre-training was performed on Jovanic *et al.* (2016) and Masson *et al.* (2020).

Of the 293 comparisons shown in the Figs 3e–g and 5a of Masson *et al.* (2020), we reproduced 232 (79%), considering a three-level outcome for the individual comparisons: positive difference, negative difference, or absence of effect. In most of the remaining cases, the MaggotUBA-based tagger led to the identification of an effect (significant difference) while the original tagger did not (218 significant differences in total versus 168, respectively). The only clear discordance (opposite effect) we found involves line *R27B12*: the proportion of *back* was expected to be significantly higher in that line than in control larvae, as reported in Masson *et al.* (2020, Fig. 5a), while the MaggotUBA-based tagging led to observing a lower proportion.

All the actions described so far (included in Fig. 2) are confirmed (*large*/*strong*) actions. Regarding the undecided (*small*/*weak*) actions, we observed that the MaggotUBA-based tagger was more perplexed overall, predicting more such small actions than the original tagger. For example, in the first 1-s window after stimulus onset, these undecided actions represented 29.9% of all the time steps with the MaggotUBA-based tagger and 18.6% with the original tagger.

The differences in behavioural readout between the taggers are most likely explained by the reliance of the MaggotUBA-based tagger on a 2-s time window only, while the original tagger considers entire tracks. In addition, the expert knowledge might be too sparse in the limited training dataset. For example, priority rules introduced in the original tagger made an action be preferably labelled as a *crawl* or *bend*, even with little certainty, rather than as a *hunch* or *roll*.

### 3.2 Transfer learning

The large amount of data accumulated at Janelia Research Campus, such as described in Vogelstein *et al.* (2014), Jovanic *et al.* (2016) and Masson *et al.* (2020), is expected to provide collections of behaviours with unprecedented diversity. Indeed, the different genetic lines of *Drosophila* exhibited differences in their resting behaviour and their responses to environmental stimuli, hence our interest in taking advantage of the information that the respective data repositories may contain.

The approach consists in pre-training the MaggotUBA autoencoder on these repositories in a self-supervised fashion (no tagging required) to extract general features of the continuous behaviour. Reusing an encoder pre-trained on large amounts of unlabelled data promotes MaggotUBA-based tagger’s ability to achieve higher accuracy when trained with relatively little annotated data [see Balestriero *et al.* (2023)].

To test this approach, we generated a new dataset in which we activated a randomly chosen set of 50 split-GAL4 lines that express the optogenetic activator of neural activity, Chrimson, in subsets of larval neurons [Meissner *et al.* (2024); see also Supplementary Section Optogenetic neural activation screen]. We tested *ca.* 60 animals from each split-GAL4/UAS-Chrimson population. We optogenetically activated neurons in each population for 15 s using 660 nm light. Further in this article, we will refer to this dataset as the ‘new activation screen’. However, it is a superset of the data used to train the reference tagger in Masson *et al.* (2020).

We pre-trained several MaggotUBA encoders on data from Jovanic *et al.* (2016) and Masson *et al.* (2020), and then trained and tested MaggotUBA-based taggers on small subsets of data from the new optogenetic screen, using the pre-trained encoder to build the MaggotUBA-based taggers. Indeed, data from Jovanic *et al.* (2016) and Masson *et al.* (2020) and the new optogenetic screen feature major dissimilarities that make them interesting candidates to investigate knowledge transferability between distinct experiments. Data from Jovanic *et al.* (2016) and Masson *et al.* (2020) consists of inactivating one or multiple neurons of the larva and characterizing the behavioural response to air puffs. On the other hand, data from the new optogenetic screen consists in activating individual or multiple neurons. Consequently, the observed behavioural responses differ in both the nature of actions evoked by the stimuli and their temporal dynamics.

We took the predictions of Pipeline_pasteur_janelia as ground truth. We evaluated transfer learning by comparing the tagging accuracy using a pre-trained encoder versus a naive Xavier-initialised encoder. Further details are given in the Transfer learning Supplementary Section.

Figure 3 shows a dramatic increase in *f*1-score with a pre-trained encoder at equal training budget. Interestingly, even the smallest datasets help train an initial tagger as long as a pre-trained encoder is used.

Without pre-training, the accuracy exhibits large variations between trials, which may result from a lack of data and insufficient training budget, especially for the larger datasets. Pre-training ensures a more stable training experience and may save time when tweaking the hyper-parameters. These results are consistent with the literature in self-supervised learning regarding accuracy improvement in downstream tasks with pre-trained neural networks. However, improvements are not systematic and were to be demonstrated in the present application. Indeed, the diversity of the behavioural phenotypes, variability in larval shapes and data quality, and the behavioural drift observed over time could have limited the generalizability of the features learned during pre-training.

In practice, the lower *f*1-scores in Fig. 3 are mainly attributable to *bends* misclassified as other actions and *vice versa*. In particular, most *rolls* were false positives. It is worth noting there are very few *rolls* in the pre-training dataset, according to experts. Still, enough could be found among Pipeline_pasteur_janelia’s predictions [see inductive bias in Blanc *et al.* (2024)]. With accumulating user experience from other experiments, training a tagger with a pre-trained encoder may suffer similar defects when the corresponding data exhibit peculiarities such as high tracking noise levels or abnormally slow larvae. In those cases, we found that the training budget should be increased so that the weights in the encoder are fine-tuned to unlearning the patterns seen in pre-training data that do not generalize well to the new training data.

As a final note, pre-training data are not required to train new taggers, whatever the action dictionary is, because LarvaTagger readily ships with a pre-trained encoder. Pre-training may be useful with other species of larvae, stages of development other than the third instar, or actions better identified over more than 2 s, for example.

## 4 Conclusion

LarvaTagger brings several improvements in *Drosophila* larval behaviour analysis, with a modular design to accommodate various tagging techniques and, at first, approach behaviour in terms of discrete actions.

Currently, it features a pre-trained deep neural network that allows training new taggers with relatively low amounts of data. The same neural network can also generate continuous representations of the behaviour, which opens a new perspective on behaviour characterization and does not heavily rely on expert annotation.

We argue this is a key advantage because behaviour is a complex concept whose definition is subject to incremental and/or contextual changes. New behaviours can emerge from new experimental paradigms, especially if behaviours are defined in terms of the function they fulfil for the behaving animal (crawling to go forward, bending to change direction, hunching to shelter the sensory centres in the head, etc.). More commonly, behaviour is first defined as patterns of movement. However, if man-made, this approach typically results in poorly defined actions, with no specifications of the initiation and termination of the behaviour. Last but not least (evolutionary biology offers a fourth approach to studying behaviour; see Tinbergen’s four questions), behaviour can also be defined mechanistically, describing how the observed movement patterns are generated. For example, if a neuro-muscular program can be unveiled, behaviour may eventually be decomposed into complex sequences of intermediate actions. Yet, the relationship between neural computation and muscle-mediated behavioural output remains to be properly modelled.

In practice, larval actions and postures have been defined in part with a focus on discriminating between these actions or postures. When asked to annotate the same data examples, the proposed definitions are sometimes too loose for multiple experts to agree on. The difficulty in formalizing and discretizing behaviour can be circumvented by taking the alternative route of data-driven behaviour analysis, paved by approaches such as MaggotUBA. The choice currently depends on the experimental paradigm and modelling goals.

At present, LarvaTagger is actively used in a study associated with a larval model of Alzheimer’s disease in *Drosophila* larvae (in preparation) and in a large-scale pesticide screening experiment (Gandara *et al.* 2024).

## Supplementary Material

btae441_Supplementary_Data

## Data Availability

The data underlying this article will be shared on reasonnable request to the corresponding author.
