# Supplementary material for: LarvaTagger: manual and automatic tagging of Drosophila larval behaviour
Source: Bioinformatics. 2024 Jul 5;40(7):btae441. doi: 10.1093/bioinformatics/btae441 (PMC11262801; doi:10.1093/bioinformatics/btae441)
Supplement: btae441_Supplementary_Data [file btae441_supplementary_data.zip › larvatagger_v2_supp.pdf]

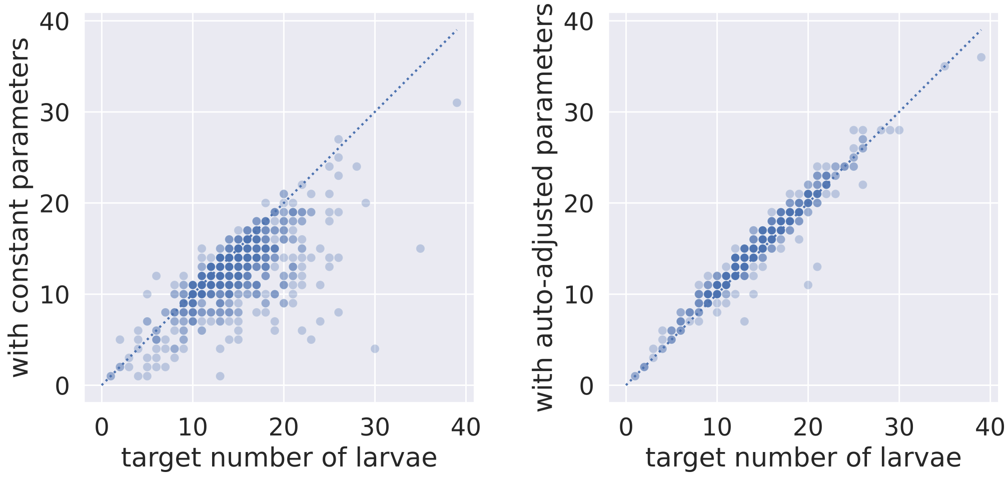

Fig. 4: Maximum number of simultaneously tracked larvae (ordinates) in every assay among 699 video files versus the known number of larvae (abscissa). Each transparent blue dot represents one or more assays. Ideally, they should align along the $x = y$ axis.

Supplementary information

Optogenetic neural activation screen

The optogenetic activation experiment referred to as the "new activation screen" was performed as previously described (Ohyama *et al.*, 2015; Jovanic *et al.*, 2016). For each behavioural assay, *Ca.* 30 larvae were separated from food by bathing them in a 20% sucrose solution for a maximum of 10 minutes. They were rinsed and placed into a square 23 cm^2 behavior rig covered with 4% agar. We recorded videos of larval behavior, with a DALSA Falcon 4M30 camera for a total of 120 s. At 30 and 75 s, 15 s-long pulses of 660 nm red light (4 µW/mm^2, Philips Lumileds) were applied.

Larvae were tracked in real-time using the Multi-Worm Tracker (MWT) software (Swierczek *et al.*, 2011; Ohyama *et al.*, 2013; Vogelstein *et al.*, 2014). We rejected objects that were tracked for less than 5s or moved less than one body length of the larva. For each larva, MWT returns contour and spine coordinates as a function of time. Raw videos are never stored.

Tracking

In this section, we briefly describe a tracking pipeline successfully applied to another large-scale screening experiment that required the fully automatic processing of thousands of 1-minute videos, recording freely moving *Drosophila* larvae exposed to different pesticides.

The pipeline is available as a Docker image and can be pulled from quay.io. The image generation code and documentation is available at gitlab.com/larvataggerpipelines/mwt-container.

This pipeline includes:

- `mwt-core`, a C++ library for online tracking that constitutes the core component of a tracking solution known as Multi-Worm Tracker or MWT (Swierczek *et al.*, 2011);
- Choreography, a Java utility (part of MWT) for offline processing of the tracks produced by `mwt-core`;
- some additional C++, Python and Julia code to load video files, using the OpenCV library, and operate `mwt-core` and Choreography in a head-less fashion (see `mwt-cli`);
- an optimisation procedure written in Python, using the Optuna library, to automatically adjust the tracking hyper-parameters so that the average number of tracked larvae approaches the target number of larvae (see `larva-tagger-tune`).

The rationale for adding the optimisation approach is that the number of larvae in the assay is always controlled or measured. If the recording parameters are stable enough, adjusting the tracking parameters may not be required. Otherwise, achieving several tracked objects similar to the known number of moving larvae can guide the automatic selection of the tracking parameters.

Fig.4 shows that in the case constant hyper-parameters do not generalise well to the whole collection of videos, the above-mentioned strategy is an efficient alternative. In the present experiment, we picked 699 videos, manually adjusted the hyper-parameters on 3 of these videos on the one hand (left panel), and automatically adjusted the hyper-parameters on a per-assay basis on the other hand (right panel).

The `20230311` tagger

Technical and methodological details of MaggotUBA are provided in Blanc *et al.* (2024).

Although LarvaTagger can support substantially different tagging backends, we highlight one particular MaggotUBA-based tagger referred to as `20230311`. `20230311` is composed of a MaggotUBA encoder with a 25-dimension output (*i.e.* 25 latent features) and a downstream classifier consisting of a single dense (fully connected) layer. It assigns each data point any of the following 7 labels: *back-up*, *bend*, *crawl*, *hunch*, *roll*, *stop* and *small action*. These labels match the terminology used in Masson *et al.* (2020) and — for users of the original tagger — are supposed to match labels from `Pipeline_pasteur_janelia` as follows:

**`20230311` labels**	**labels in trx.mat files**
back-up	*back_large* = *back_strong*
bend	*cast_large* = *cast_strong*
crawl	*run_large* = *run_strong*
hunch	*hunch_large* = *hunch_strong*
roll	*roll_large* = *roll_strong*
stop	*stop_large* = *stop_strong*
small action	*small_motion*

A valid data point is a 2-s time segment of tracking data, can be taken at any defined time step with at least 1 s of past and future history, and consists of a time series of 5-point spines (or mid-lines) re-sampled at 10 Hz around the time step of interest. For prediction tasks, data points in a track's first or last seconds are labelled in a nearest-neighbour fashion.

As a first step, an autoencoder was trained with self-supervision on a subset of combined data from the new optogenetic screen described in section **Transfer learning** and the screen from Jovanic *et al.* (2016); Masson *et al.* (2020). No labelling information was used except for the inductive bias in the pre-training dataset formation that consisted of randomly picking data examples of the different actions so that no classes (or actions) were represented more than twice the least common class. This $\times 2$ limitation made the pretraining dataset small in comparison with the training dataset, but ensured rare events such as *rolls* were almost equally represented.

In a second step, the resulting pre-trained encoder was combined with the classifier and a new training dataset was sampled from the new optogenetic screen, involving 1 200 235 data examples. These data examples were randomly picked so that no classes were represented more than 20 times the least common class. The combined encoder+classifier was trained with a cross-entropy loss and a 10 000-iteration budget. In the first 5 000 iterations, the classifier only was updated. The encoder and classifier were fine-tuned in the remaining 5 000 iterations.

Similarly to MaggotUBA, training parameters were stored in (2) JSON files and the learned weights in (2) PyTorch PT files.

More details can be found in gitlab.pasteur.fr/nyx/MaggotUBA-adapter#20230311-0-and-20230311.

Evaluation on data from Jovanic et al. (2016); Masson et al. (2020)

The analysis is demonstrated in a code repository available at gitlab.com/larvataggerpipelines/t5_analysis_replicates, in the shape of Pluto notebooks (in Julia).

The same part of the Jovanic *et al.* (2016); Masson *et al.* (2020) data repository was used as in Masson *et al.* (2020), specifically, lines obtained by crossing with the `UAS_TNT_2_0003` effector, and behavioural assays with a 30-s air-puff at $t = 45$ followed by a sequence of 10 2-s air-puffs every 10 s starting from $t = 105$.

A variant of `20230311`, available in MaggotUBA-adapter as `20230311-0`, was applied and compared with `Pipeline_pasteur_janelia`. `20230311-0` is the same tagger as `20230311`; the only difference is a post-processing step in `20230311` that maps the original 12-class output of `20230311-0` (*back_strong*, *back_weak*, *cast_strong*, *cast_weak*, etc) onto 7 classes with *weak* actions pooled together in a *small action* class, and some actions renamed (see label table 8).

The tracking data were extracted from *trx.mat* files generated by `Pipeline_pasteur_janelia`, instead of the original files generated by Choreography. Indeed, `Pipeline_pasteur_janelia` includes a head-tail correction preprocessing step, which technically is a tracking task. This correction step fixed the orientation of roughly 10% of the larvae in the Jovanic *et al.* (2016); Masson *et al.* (2020) experiment, due to the high numbers of simultaneously behaving larvae and higher probability of U-turn. To better characterise the tagging performance *per se*, we have considered this correction mechanism as common ground for comparing between taggers.

For completeness, running the same analysis on the original data files with head-tail orientation as inferred by MWT+Choreography led to a 10-point increase in baseline level of *back-up* in control larvae, for example. Although the qualitative differences reported in tables figs. 3e, 3f, 3g and 5a in Masson *et al.* (2020) were similarly preserved overall, the higher baseline level of *back-up* probability had no additive effects on known events of higher *back-up* probability. For example, in fig.2b, a 10% plateau can be seen after the stimulus onset, for example, at $t = 60$. The difference in probability between $t = 40$ to $t = 60$ disappeared which — considering the nature of the analyses that can be carried out on such a readout — is a major difference.

Transfer learning

Pre-training MaggotUBA autoencoders was performed similarly to `20230311`, here on Jovanic *et al.* (2016); Masson *et al.* (2020) only, with different latent dimensionalities (25, 50, 100, 200). Training datasets were drawn from the new optogenetic screen, randomly picking 1 000 data files and then a fraction of the available time segments in these files. The corresponding test datasets consisted of similarly sampled time segments from 100 other data files.

The training was performed with a 1 000-iteration budget (default value in MaggotUBA-adapter, subject to changes). The untrained encoders were trained together with the classifier at all iterations. In contrast, with pre-trained encoders, the budget was split in two, with 100 iterations to train the classifier initially and 900 iterations to fine-tune the encoder and the classifier jointly.

Fig.3 shows results for a 25-dimension latent space. Very little variation was observed between the explored dimensionalities. An optimum was observed with 50 dimensions.

The source code for transfer learning is available at gitlab.com/larvataggerpipelines/Autoencoding.
