# Supplementary figures and images for: LarvaTagger: manual and automatic tagging of Drosophila larval behaviour
Source: Bioinformatics. 2024 Jul 5;40(7):btae441. doi: 10.1093/bioinformatics/btae441 (PMC11262801; doi:10.1093/bioinformatics/btae441)

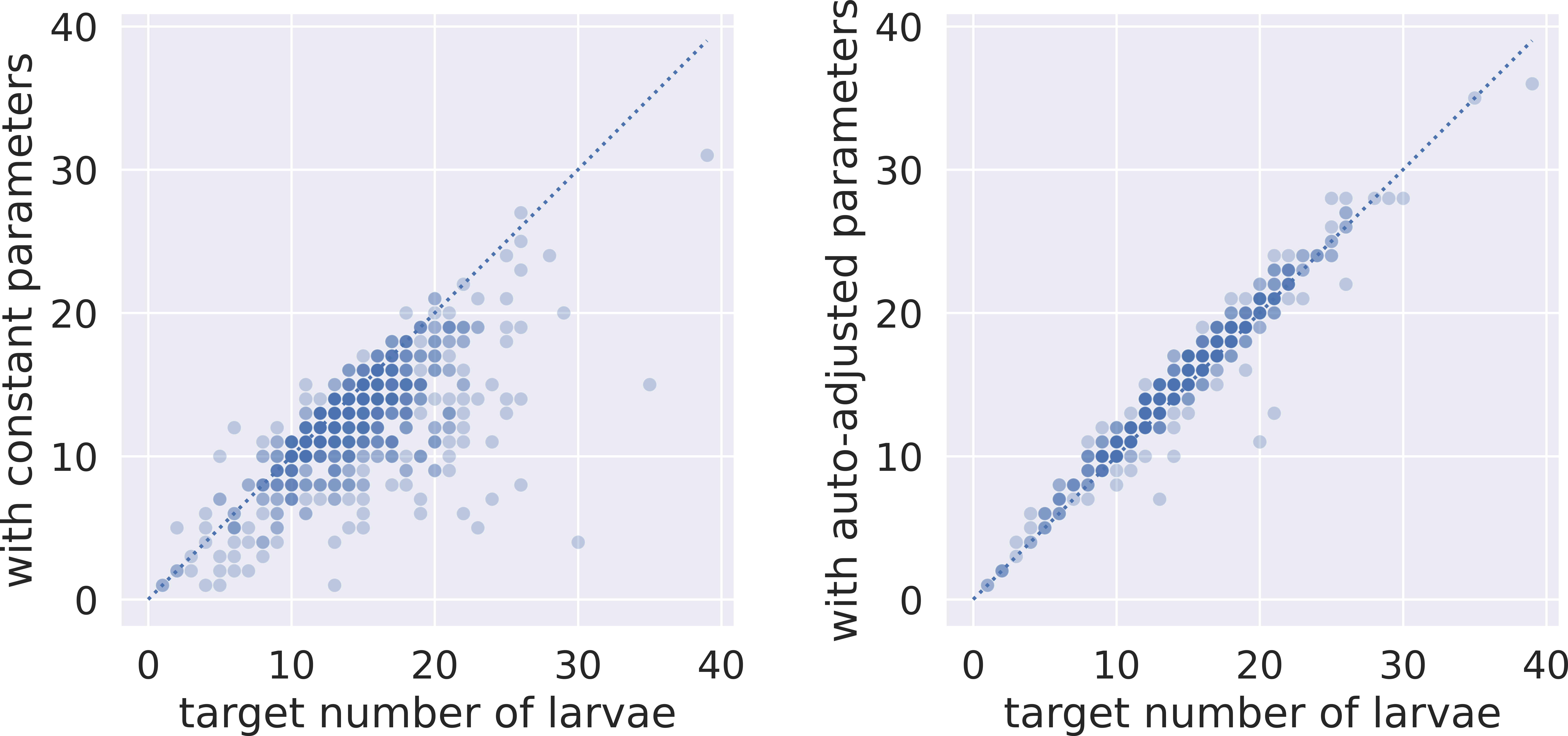

Supplement: btae441_Supplementary_Data [file btae441_supplementary_data.zip › supp_fig1.png]
